# hZip2 and hZip3 zinc transporters are down regulated in human prostate adenocarcinomatous glands

**DOI:** 10.1186/1476-4598-6-37

**Published:** 2007-06-05

**Authors:** Mohamed M Desouki, Joseph Geradts, Beatrice Milon, Renty B Franklin, Leslie C Costello

**Affiliations:** 1 Dept of Pathology and Lab Medicine, Medical University of South Carolina, Charleston, SC, USA; 2 Department of Pathology, Duke University Medical Center; Durham, NC, USA; 3 Department of Biomedical Sciences/Dental School and The Greenebaum Cancer Center, University of Maryland, Baltimore, Maryland, USA

## Abstract

**Background:**

The normal human prostate glandular epithelium has the unique function of accumulating high levels of zinc. In prostate cancer this capability is lost as an early event in the development of the malignant cells. The mechanism and factors responsible for the ability of the normal epithelial cells to accumulate zinc and the loss of this capability in the malignant cells need to be identified. We previously reported that Zip1 is an important zinc uptake transporter in prostate cells and is down regulated in the malignant cells in situ along with the depletion of zinc levels. In this report we investigated the expression of two other Zip family zinc transporters, Zip2 and Zip3 in malignant versus nonmalignant (normal and BPH) glands. Zip2 and Zip3 relative protein levels were determined by immunohistochemistry analysis of human prostate tissue sections.

**Results:**

Normal and BPH glandular epithelium consistently exhibited the strong presence of both Zip 2 and Zip3; whereas both transporters consistently were essentially non-detectable in the malignant glands. This represents the first report of the expression of Zip3 in human prostate tissue; and more importantly, reveals that ZiP2 and Zip3 are down regulated in malignant cells in situ as we also had demonstrated for Zip1. Zip2 and Zip3 transporter proteins were localized predominantly at the apical cell membrane, which is in contrast to the Zip1 localization at the basolateral membrane. Zip2 and Zip3 seemingly are associated with the re-uptake of zinc from prostatic fluid.

**Conclusion:**

These results coupled with previous reports implicate Zip2 and Zip3 along with Zip1 as important zinc uptake transporters involved in the unique ability of prostate cells to accumulate high cellular zinc levels. Zip1 is important for the extraction of zinc from circulation as the primary source of cellular zinc. Zip 2 and Zip3 appear to be important for retention of the zinc in the cellular compartment. The down regulation of all three transporters in the malignant cells is consistent with the loss of zinc accumulation in these cells. Since zinc imposes tumor suppressor effects, the silencing of the gene expression for these transporters is a required event for the manifestation of the malignant activities of the neoplastic cells. This now provides new insights into the genetic/molecular events associated with the development of prostate cancer; and supports our concept of Zip1, and now Zip2 and Zip3, as tumor suppressor genes and zinc as a tumor suppressor agent.

## Background

It has long been established that the normal human prostate gland has the major function and capability of accumulating uniquely high levels of zinc; generally about ten-fold greater than other soft tissues. This capability resides within the glandular secretory epithelial cells of the peripheral zone, which is the major site of origin of most prostate malignancy. Over fifty years of clinical studies have consistently demonstrated that prostate cancer tissue samples consistently contain about 65% less zinc than normal prostate tissue. More precisely, the zinc concentration (nmols/gram wet weight) of normal peripheral zone tissue approximates 3,000–4,500; malignant peripheral zone tissue approximates 400–800; and other soft tissues approximate 200–400. Consequently, malignant prostate tissue zinc levels are decreased by ~70–85% compared to normal peripheral zone; and the decrease is observed in the glandular epithelial cells. Most importantly, one rarely, if ever, finds malignant glands that have retained the high zinc levels that characterize the normal gland. In addition, the decrease in zinc occurs early in the development of prostate malignancy. These established clinical relationships have raised important issues that relate to the role and mechanisms of zinc accumulation in the normal functioning of the prostate gland, and the loss of zinc accumulation as a requirement in the development of prostate malignancy. For expanded background discussion and review of these relationships see our recent papers [1-3].

The accumulation of zinc results in several important effects on prostate cells. The functional role of zinc accumulation is to inhibit citrate oxidation of the highly specialized secretory epithelial cells, which permits the production and secretion of extremely high levels of citrate as a major component of prostatic fluid. In addition to its functional role, the accumulation of zinc has consequential effects that include: metabolic effects such as inhibition of terminal oxidation, truncation of the Krebs cycle, decreased energy production; growth/proliferation inhibition effects such as induction of mitochondrial apoptogenesis; and inhibition of invasiveness and motility. The combination of such effects can be characterized as anti-tumor effects, which lead us to propose that zinc is a tumor-suppressor agent against prostate cancer. This provides the explanation for the requirement that malignant cells lose the capability to accumulate zinc and the basis for the absence of malignant glands that retain high levels of zinc.

This has led us to pursue the critical issues regarding the mechanism of zinc accumulation in the normal epithelial cells and the mechanism of the lost ability of the malignant cells to accumulate zinc. The members of the Zip family of zinc transporters have been identified as important zinc transporters for the cellular uptake and accumulation of zinc in mammalian cells. More specifically, we have identified Zip1 as an important zinc uptake transporter in prostate cells [4,5]. Our recent report [6] demonstrated that Zip1 is expressed in human normal and hyperplastic prostate glandular epithelium; and is down-regulated in adenocarcinomatous glands. Correspondingly, the high cellular zinc level is markedly depleted in the malignant glands. In addition Rishii et al [7] identified the down regulation of Zip1 expression in the high prostate cancer at-risk African American male population as compared with Caucasians. These relationships cause us to propose that Zip1 is a candidate tumor suppressor gene in prostate cancer. However, the expression and role of other Zip-family transporters in prostate malignancy have not been established. In this report we identify by immunohistochemistry the expression of Zip2 and Zip3 in non-malignant prostate tissue sections and their down-regulation in malignant prostate glands. The potential relevance of these zinc transporters in normal and malignant prostate is described.

## Results

**Figure 1:**
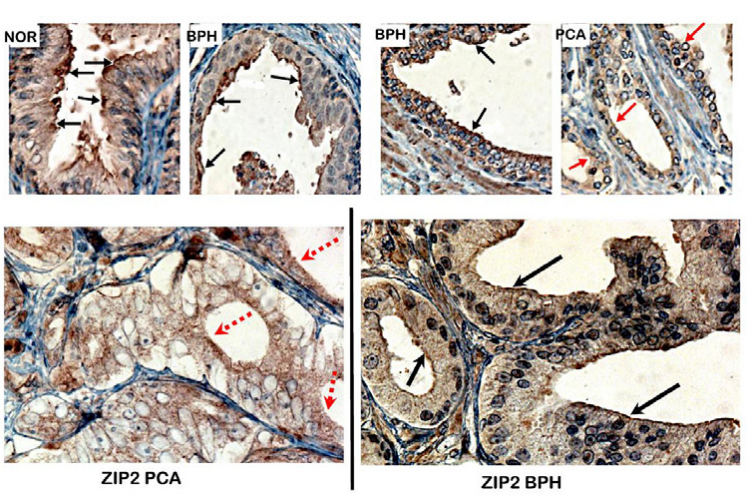
Immunohistochemical detection of Zip2 transporter protein in normal, BPH and malignant sections of representative prostate tissues. Note the immuno-positivity of the apical plasma membrane of normal and BPH glands (black arrows). The malignant glands show no detectable Zip2 membrane immunoreactivety (red arrows).

**Figure 2:**
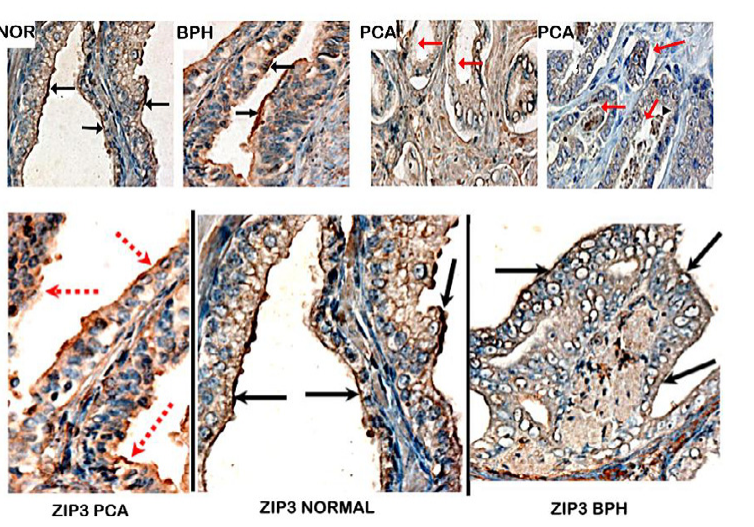
Immunohistochemical detection of Zip3 transporter protein in normal, BPH and malignant sections of representative prostate tissues. (top; magnifications are ×400). Note the immuno-positivity of the apical plasma membrane of normal and BPH glands (black arrow). The malignant glands show no detectable Zip3 membrane immunoreactivety (red arrows).

Figures 1 and 2 show representative results of the Zip2 and Zip3 immunohistochemical staining observed in normal, BPH glands, and adenocarcinomatous glands, respectively. The glandular epithelium of the normal and BPH glands exhibits immuno-positive Zip2 and Zip3, both of which exhibit staining that is localized predominantly at the apical cell membrane of the secretory epithelium. In contrast, in the adenocarcinomatous glands both transporters are negligible to absent in the malignant cell membranes. It is also evident that both Zip transporter proteins studied are minimally detected in the stromal tissue.

Tables 1 and 2 provide the summary of the comparative immunohistochemical scoring of hZip2 and Zip3 reactivity of normal, BPH, and malignant prostate glands respectively. Normal glands and BPH glands are both zinc-accumulating glands as contrasted with malignant glands that do not accumulate high zinc levels. Only 4 of 30 combined normal plus BPH cases were negative for Zip2, compared to 21 of 24 malignant cases being negative. None of the 30 normal plus BPH cases was negative Zip3, compared to 21 of 24 malignant cases being negative. The results clearly establish that both Zip2 and Zip3 are down regulated in malignant prostate glands

**Table 1:** Zip2 immunopositivity of glandular components in prostate tissue sections.

**Case no^**a**^.**	**Zip2 IHC SCORE**^**b**^		
	**Normal**	**BPH**	**Malignant**
**1**	**+++**	**+++**	**-**
**2**	**N/A**	**++**	**-**
**3**	**N/A**	**++**	**-**
**4**	**N/A**	**++**	**-**
**5**	**N/A**	**++**	**-**
**6**	**+++**	**+++**	**-**
**7**	**+++**	**++**	**+**
**8**	**N/A**	**++**	**++**
**9**	**++**	**+**	**-**
**11**	**N/A**	**++**	**-**
**12**	**N/A**	**+**	**-**
**13**	**N/A**	**++**	**-**
**14**	**N/A**	**+**	**-**
**15**	**N/A**	**+**	**-**
**16**	**N/A**	**-**	**-**
**17**	**N/A**	**-**	**-**
**18**	**N/A**	**+++**	**-**
**20**	**N/A**	**-**	**-**
**21**	**N/A**	**++**	**-**
**22**	**N/A**	**+++**	**-**
**23**	**N/A**	**-**	**-**
**24**	**N/A**	**+++**	**N/A**
**25**	**++**	**++**	**-**
**26**	**N/A**	**++**	**-**
**27**	**N/A**	**+++**	**+**

**Neg Zip2 IHC**	**0/5**	**4/25**	21/24*
**Scores > +**	**5/5**	**17/25**	1/24*
**Mean score**^**c**^	**2.60**	**1.76**	0.17*

^a^Case number: Cases 1–23 are the same cases from our previous studies [6,8]. Cases from 24–27 are new cases.

^b^Scoring of immunoreactivity was done as follows: negative, no positive cells; score +, < 10% positive cells; score ++, 10–50% positive cells; score +++, > 50% positive cells.

^c^Mean score: Mean for each group was obtained by the sum of the +'s/number of cases.

*P < 0.001 for malignant vs normal or vs BPH.

**Table 2:** Zip3 immunopositivity of glandular components in prostate tissue sections

**Case no^**a**^.**	**Zip3 IHC SCORE**^**b**^		
	**Normal**	**BPH**	**PCa**
**1**	**+**	**+**	**-**
**2**	**N/A**	**+**	**-**
**3**	**N/A**	**+++**	**-**
**4**	**N/A**	**+**	**-**
**5**	**N/A**	**++**	**+**
**6**	**++**	**++**	**-**
**7**	**++**	**+**	**-**
**8**	**N/A**	**++**	**+**
**9**	**++**	**++**	**-**
**11**	**N/A**	**+++**	**-**
**12**	**N/A**	**++**	**-**
**13**	**N/A**	**++**	**-**
**14**	**N/A**	**+**	**+**
**15**	**N/A**	**+**	**-**
**16**	**N/A**	**+**	**-**
**17**	**N/A**	**++**	**-**
**18**	**N/A**	**+++**	**-**
**20**	**N/A**	**+++**	**-**
**21**	**N/A**	**++**	**-**
**22**	**N/A**	**+++**	**-**
**23**	**N/A**	**+**	**-**
**24**	**N/A**	**+++**	**N/A**
**25**	**+++**	**+++**	**-**
**26**	**N/A**	**++**	**-**
**27**	**N/A**	**+++**	**-**

**Neg Zip3 IHC**	**0/5**	**0/25**	21/24*
**Scores > +**	**4/5**	**17/25**	0/24*
**Mean score**^**c**^	**2.00**	**2.00**	0.13*

See Table 1 for footnotes

The down regulation of Zip2 and Zip3 is nearly identical to the down regulation of Zip1 that we had previously reported [6]. Table 3 shows the composite results from this study and our previous study of Zip1 for the three transporters in identical subjects. Of the twenty-one common tissue samples from subjects, thirteen showed negativity of all three transporters, and seven cases showed either negative or low immunopositivity (+) for all three transporters in the malignant glands. Therefore, twenty of the twenty-one cases showed negative or lowest level of immuno-positivity for all three transporters. This reveals the consistency of down regulation of Zip transporters in malignant glands. In addition, we earlier reported that m-aconitase IHC performed with the same samples represented in Table 3 showed no difference between the malignant and BPH glands [8]. This reveals the specificity of the down regulation of the Zip transporters. Also as shown in Table 3, there exists no correlation between the PCA scoring and the grade of cancer for any of the transporters. This is consistent with our contention that the zinc and citrate metabolic changes occur in the early stage of the development of malignancy (perhaps premalignant stage) and persists through the progression of malignancy; and this would also apply to the down regulation of the Zip transporters [1-3,6].

**Table 3:** Comparative results for Zip1, Zip2, Zip3, and m-aconitase

**Case**	**GRADE^**	**Zip2**	**Zip2**	**Zip3**	**Zip3**	**Zip1**	**Zip1**	**K^^**	**ACON**	**ACON**
		**BPH**	**PCA**	**BPH**	**PCA**	**BPH**	**PCA**		**BPH**	**PCA**
**1**	**3**	**+++**	**-**	**+**	**-**	**+++**	**-**	*	**+++**	**++**
**2**	**3**	**++**	**-**	**+**	**-**	**++**	**+**	**	**+**	**+++**
**3**	**1**	**++**	**-**	**+++**	**-**	**+**	**-**	*	**+**	**+**
**4**	**2**	**++**	**-**	**+**	**-**	**+**	**-**	*	**+++**	**+++**
**5**	**2**	**++**	**-**	**++**	**+**	**-**	**-**	**	**++**	**+**
**6**	**1**	**+++**	**-**	**++**	**-**	**+++**	**-**	*	**+++**	**+++**
**7**	**2**	**++**	**+**	**+**	**-**	**+**	**+**	**	**++**	**+++**
**8**	**1**	**++**	**++**	**++**	**+**	**+++**	**+**		**+++**	**++**
**9**	**1**	**+**	**-**	**++**	**-**	**+++**	**-**	*	**+**	**+++**
**11**	**2**	**++**	**-**	**+++**	**-**	**++**	**-**	*	**+**	**++**
**12**	**1**	**+**	**-**	**++**	**-**	**-**	**-**	*	**+**	**+++**
**13**	**2**	**++**	**-**	**++**	**-**	**++**	**-**	*	**+++**	**+++**
**14**	**1**	**+**	**-**	**+**	**+**	**+**	**-**	**	**+++**	**+++**
**15**	**1**	**+**	**-**	**+**	**-**	**+**	**-**	*	**+**	**+**
**16**	**1**	**-**	**-**	**+**	**-**	**+**	**-**	*	**++**	**+++**
**17**	**1**	**-**	**-**	**++**	**-**	**+++**	**+**	*	**++**	**-**
**18**	**2**	**+++**	**-**	**+++**	**-**	**++**	**+**	**	**++**	**+++**
**20**	**1**	**-**	**-**	**+++**	**-**	**+**	**-**	**	**+++**	**++**
**21**	**1**	**++**	**-**	**++**	**-**	**+++**	**+**	*	**+++**	**+++**
**22**	**1**	**+++**	**-**	**+++**	**-**	**-**	**-**	*	**+++**	**+++**
**23**	**2**	**-**	**-**	**+**	**-**	**ND**	**ND**	**	**+++**	**+++**
**MEAN SCORE**		**1.6**	**0.I4$**	**1.85**	**0.14$**	**1.28**	**0.33$**		**2.09**	**2.38$$**

^PCA grading described in reference [6].

^^K = Constant result for PCA scores of Zip1, Zip2, Zip3

* all are negative in PCA;

** all are negative or + in PCA

$ = P < 0.001 PCA vs BPH; $$ = No significant difference PCA vs BPH

Zip1 results taken from reference [6].

Aconitase results taken from reference [8].

ND = not determined

**Figure 3:**
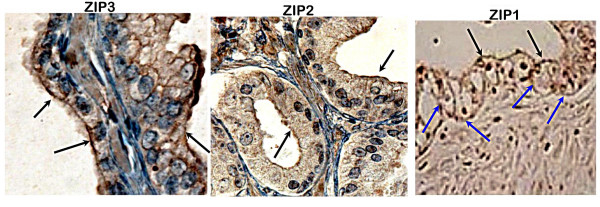
Comparative localization of Zip1, Zip2, and Zip3 in prostate glands. The black arrows point to the immunopositive staining at the apical membrane. The blue arrows point to the immunopositive staining at the basoloateral membrane.

However the results also reveal an important difference between Zip 2 and Zip3 compared to Zip1. Figures 1 and 2 show that Zip2 and Zip3 transporters are localized nearly exclusively at the apical membrane. This is in contrast to our earlier report that showed the dominant localization of Zip1 at the basolateral membrane. This is further revealed in figure 3, which shows the definitive Zip1 immunostaining of the basolateral membrane and the apical membrane of the normal prostate glandular epithelial cells. Note also the absence of Zip1 in the stroma. The limitation of Zip2 and Zip3 to the apical surface raises important issues concerning the functional role of these transporters (discussed below).

## Discussion

This report coupled with our earlier study reveals that Zip1, Zip2, and Zip3 are expressed in situ in normal prostate glandular epithelial cells and also in hyperplastic glandular epithelium; both of which are zinc-accumulating glands. This is the first report that identifies the expression of Zip3 in human prostate glands. In contrast, the expression of all three transporters is consistently down-regulated in adenocarcinomatous glands, which are known to have lost the ability to accumulate zinc. These results coincide with the numerous reports that have established the consistently marked decrease in the zinc levels of prostate cancerous tissue as compared to normal and BPH prostate tissue. Thus, the expression of these Zip family zinc uptake transporters in normal and BPH glands and the loss of their expression in malignant glands are further evidence of their role in the cellular accumulation of zinc, or lack thereof, in prostate cells. It is notable that Rishii et al [7] reported the down regulation of expression of Zip 2 as well as Zip1 in African-American males.

The localization of Zip1 at the basolateral membrane of normal glandular epithelial cells is consistent with its cellular function to transport zinc from the interstitial fluid derived from blood plasma into the cell. Therefore, Zip1 provides the source of the cellular zinc. Correspondingly, the down regulation of Zip1 in malignant cells is consistent with their inability to accumulate zinc. However the present study shows that Zip2 and Zip3, unlike Zip1, exhibit little or no localization at the basolateral membrane, and are confined predominantly to the apical membrane. Studies with cell lines have shown that Zip2 and Zip3 function as zinc uptake transporters [9,10]. Therefore the in situ apical membrane localization indicates that Zip 2 and Zip3, unlike Zip1, do not function to accumulate cellular zinc from circulation. If one presumes that they retain zinc uptake transporter activity in situ, their functional role might be to conserve the cellular zinc level by transporting (reabsorbing) zinc from the prostatic fluid back into the epithelial cell. Normal prostatic fluid has a high zinc concentration of ~9 mM compared to interstitial fluid zinc level of ~7 uM. At this high prostatic fluid concentration and depending upon the Km value of each transporter for zinc, Zip2 and Zip3 might effectively contribute to maintaining a high intracellular level of zinc. This implies that the down-regulation of Zip2 and Zip3 in the malignant cells would contribute to their loss of cellular zinc by the elimination of the "reabsorption" of zinc from prostatic fluid. However, it is Zip1 that is the major factor since its down regulation eliminates the principal source of cellular zinc; namely zinc uptake from circulation. Presently we can only speculate regarding the role of Zip 2 and Zip3 in zinc accumulation by normal prostate glands in situ; and the possible implications of their down regulation in the malignant glands. It is also notable that Rishii et al [7] had reported that Zip1 and Zip2 mRNA analysis revealed their expression in normal prostate glands of Caucasians, but their expression was down regulated in Black Americans; which appeared to correlate with the higher incidence of prostate cancer among this population.

An important issue is the mechanism that is responsible for the down regulation of the Zip transporters. Because Zip1 expression reappears in several immortalized prostate cancer cell lines under standard culture conditions, we have proposed that its down regulation in situ results from the epigenetic silencing of gene expression [1-3,6]. Similarly, both Zip2 and Zip3 are down regulated in the primary site malignant glands in situ, but their expression re-appears in the immortalized prostate cancer cell lines: Zip3 in RWPE2 cells [9]; Zip2 in PC-3 cells [our unpublished studies]. This leads us to propose that their in situ down regulation also results from epigenetic effects. Presently, no information exists regarding the expression of any of these transporters in metastatic cells in situ. It will be critically important to establish if the down regulation in the primary site malignant cells is retained after the cells intravasate and develop as metastatic cells in distant tissue sites. This will provide key information regarding important in situ factors that are involved in the regulation of these important potential tumor-suppressor genes. We are currently pursuing such studies.

We have proposed that the various effects of zinc in prostate cells constitute tumor-suppressor actions. The functional role of zinc is its prevention of citrate oxidation that is achieved by its inhibitory actions on m-aconitase activity [11,12] of the highly specialized citrate-producing prostate cells. This capability now appears to result from the expression of the Zip zinc uptake transporters that maximize the accumulation of cellular zinc. The inhibition of citrate oxidation and truncation of the Krebs cycle imposes bioenergetic and metabolic conditions under which most mammalian cells cannot survive [1-3]. The high zinc levels also impose other consequential adverse effects. Zinc induces mitochondrial apoptogenesis and other effects that inhibit growth and proliferation [1-3,10,13-15]. Obviously, the normal glandular epithelial cells under in situ conditions have evolved with mechanisms and adaptations that prevent these adverse effects. Apparently the neoplastic malignant cell is susceptible to these adverse effects of high cellular zinc. In addition, zinc also imposes inhibitory effects on mobility and invasiveness of malignant prostate cells [16,17]. To avoid these effects, the neoplastic cell decreases its cellular zinc level by down regulation of the Zip zinc uptake transporters, which is why malignant prostate glands virtually always exhibit low zinc levels in contrast to the high zinc levels that characterize normal prostate glands. In the absence of high zinc levels, the synthetic, bioenergetic, growth and proliferation, and invasive requirements of malignancy can be manifested. Consequently we propose that zinc is a tumor-suppressor agent; and Zip1, along with Zip2 and Zip3, is a tumor suppressor gene in prostate cancer.

## Conclusion

Immunohistochemical examination of human prostate tissue sections reveals the existence of the zinc uptake transporters hZip2 and hZip3 in the zinc-accumulating normal and BPH human prostate glandular epithelium. Most importantly, we also show that both hZip2 and hZip3 are markedly down regulated in adenocarcinomatous glands, which have lost the ability to accumulate zinc. These results are similar to earlier observations with hZip1. Unlike hZip1, hZip2 and hZip3 transporters are confined to the apical membrane with little or no demonstrable transporter protein evident at the basolateral membrane. hZip2 and hZip3 appear to function for the re-uptake and conservation of zinc from prostatic fluid, and hZip1 functions for the accumulation of zinc from circulation. This combination optimizes the cellular accumulation of zinc in normal prostate glands. Since zinc accumulation exhibits tumor suppressor actions, the down regulation of these transporters in the malignant cells is essential for the cellular depletion of zinc to prevent the anti-tumor effects of zinc. These findings are consistent with our concept that Zip1, and now Zip2 and Zip3, are tumor-suppressor genes in prostate cancer; and provide the explanation and genetic/molecular/metabolic mechanism for the virtual absence of malignant prostate glands that retain the high zinc levels that characterize the normal glands.

## Methods

### Human tissue samples

Formalin fixed paraffin embedded prostatic tissue sections were used in this current study. The studies were conducted with twenty-five prostatic adenocarcinoma cases of which twenty-four contained both BPH and adenocarcinomatous foci, and five cases also showed normal prostatic acini. The cases numbered 1–23 were the same samples that were used for Zip1 and m-aconitase immunohistochemistry studies presented in our earlier reports [6,8], and cases numbered 24–27 are new samples. The immunohistochemical analysis of the prostate tissue sections were conducted without the identification of the subjects involved in study.

### Immunohistochemistry of human prostatic tissue sections

Immunohistochemistry with anti-hZip2 and anti-hZip3 antibodies was performed as described [6] with modification. Briefly, the slides were deparaffinized by heating at 60 degrees for one hour followed by xylene and ascending grades of alcohol washes. Antigen retrieval was done by heating in Dako sodium citrate buffer at 98°C. Dako Autostainer programmed as follows; 30 min with Blocking Buster, 1 hour with either 1.2 ug/ml of anti-Zip2 or 0.8 ug/ml of anti-Zip 3 diluted in Dako antibody diluent, followed by incubation with anti chicken IgY HRP conjugate rabbit secondary antibody in a dilution of 1:200 (Promega, cat # G1351) for 30 minutes. Color was developed by incubating slides with DAB+ Chromogen (Dako, Code # K4007) followed by Hematoxylin counterstaining. Sections were examined with light BX40 Olympus Microscope. Pictures were taken with digital camera. The appearance of membrane-associated immuno-positivity for Zip2 and Zip3 of the glandular epithelial cells were used for scoring as previously described [6,18]. The scores employed were; negative, no positive cells; + < 10% positive cells; ++ 10–50% positive cells; +++ > 50% positive cells. The mean scores between groups were analyzed by the Student's t-Test

## Competing interests

The author(s) declare that they have no competing interests.

## Authors' contributions

LCC and RBF conceived the concept, designed the study, and prepared the manuscript. BM developed and tested the antibodies employed in the study. MD provided the human tissue samples. MD and JG performed the immunohistochemical and pathological analyses of the human tissue samples and provided the data. All authors have read, edited, and approved the manuscript.
